# London's Great Problem Solved

**Published:** 1887-12-17

**Authors:** 


### LONDON'S GREAT PROBLEM SOLVED.

(*Continued from No.* 62, *Vol. iii.*)

### II.—HOW EACH METROPOLITAN CONSTITUENCY MAY PERMANENTLY PROVIDE FOR THE NEEDS OF ITS CASUAL AND UNEMPLOYED POOR.

IN proceeding to give an outline of the scheme we propose for providing an effectual remedy for the distress, whether acute or chronic, which exists amongst the population of each metropolitan constituency, it may be well at the outset to briefly set forth the views held on the general subject. The cause of much of the distress which prevails in London is due to the fact that residents in the metropolis have not the same touch with and knowledge of their poorer neighbours as exists in provincial towns and smaller communities. It seems essential to recognise this fact at once, and to realise that with the exception of certain well-organised parishes we, at present, possess no precise knowledge of the extent of the distress which exists. If this view be correct, the true remedy will be found in a new departure which aims at obtaining precise and accurate information and knowledge of the needs and circumstances of every poor family and person resident in each constituency, both at the present time and henceforward for all time. Having studied this question in all its bearings for many years, we believe that the City of Boston, in the United States, has organised a system which, with modifications, is best adapted to the requirements of every constituency throughout London. Our proposals are based upon a personal knowledge of the Boston system and its results, and we are indebted to the publications of the Associated Charities of Boston for much which follows. We must bear in mind that among the pauperised class of a great city, the chief obstacles are two, usually found together: lack of all skill, and lack of all hope. They can do nothing well enough to get work, and they are sunk in despair. They will make no effort to help themselves, or if you succeed in inducing them to try, there is so little they can do! Leave them as they are, and they sink lower. Their children grow up in the midst of pauperism, expecting, if not even preferring, to be paupers like their parents. Industrial training-schools are a potent remedy, but further agencies for training women are needed. Chief among them should be an Industrial Home in every constituency, with its laundry, its rooms where women are taught sewing, cutting, and machine-work, its carpentering for boys, with a *crèche* for infants. The laundry would soon be able to point to many poor women now earning their own support, who a year before were dependent on alms. The relations growing out of teaching these poor women would enable a visitor to gain a friendly and powerful influence over them, inspiring them with new hope, and confidence, and self-respect.

### FIRST A LITTLE SKILL AND A NEW HOPE, AND THEN?

Thus, a certain degree of skill, and a new hope, would replace the old ignorance and despair, and so the two most fatal obstacles we have to face would often be overcome. We are inclined to think that volunteer visiting is the only hope of civilisation against the gathering curse of pauperism in great cities. Many proposals are always made by local committees for affording relief to the distressed by way of loans, or by donations to existing charities, to be distributed at their discretion. Taking the last proposal first, we object altogether to its adoption by any local committee as a panacea for the existing distress, for the reason that it would enable us to shirk our plain duty, which is to face and so to avoid the difficulties presented by the existing distress in every constituency, and then, if possible, to find out how that distress can be effectually and permanently treated upon some method which offers hope of permanent alleviation, if not of total removal.

### A GIFT OR A LOAN.

Let us next consider if the aid should be a gift or a loan. If a loan, we should be sure that there are nine chances to one in favour of the applicant repaying it, else we encourage a breach of faith, that is worse than the effect of a direct gift. If a gift, let it be enough, and so applied as to put the family on a permanent footing of self-support. Small and uncertain doles are ruinous to the recipients. In chronic cases it is desirable to obtain a pension in money or kind for a similar reason. In cases which will be "chronic" until children grow up, let us arrange, if possible, and make the parents understand that as each child becomes of working age, the pension or allowance will be reduced; and when the earnings are large enough, aid will be withdrawn altogether.

### OTHER PROPOSALS AND WHAT THEY LEAD TO.

Another proposal often is that workmen should be provided with tools and clothes, where such help is found to be calculated to permanently benefit the recipients. Emigration for certain special cases is also generally favoured; and there is an unanimous desire to arrange, if possible, that suitable work shall be provided for the unemployed, who are genuinely anxious to obtain it. Now why, it may be asked, do we recite the opinions in this systematic manner? To show to every thoughtful reader that we are powerless, and must remain powerless to wisely and fully carry out any one of these proposals, unless or until we have an accurate knowledge of the nature and extent of the distress to be relieved, and of the numbers and circumstances of the unemployed whom it is desirable to provide with suitable work. In brief, we have at present no machinery or organisation in any London constituency to enable us to do this work effectually, wisely, and completely, and so our first step must be to supply it, if we are ever to secure the end in view.

### First Register all Relief Agencies and the Unemployed.

We would therefore suggest that we should take immediate steps to obtain precise and accurate information—

I. Of all the relief given in each Metropolitan constituency by the Poor-law Authorities and the various charities; that is, that a Register of all the relief given shall be opened at once.

II. Of all the men and women who are out of work, and who desire to obtain it; that is, a register of the unemployed and a labour bureau.

When we consider how many charitable societies, as well as numerous Church Benevolent Societies, are now engaged in distributing money and aid of various kinds, and that many of them are without exact knowledge of the specific objects or the actual work of the rest, no further proof is needed to show that the very relief they aim to afford cannot be satisfactorily given, for they must, more or less, overlap one another, and waste of time, strength, and money be the necessary result. We believe that greater familiarity with each other's work would tend to diminish imposture and street begging, which all are desirous to prevent, and thus greatly benefit the poor. We would therefore propose to open an office in each constituency to which all societies and individuals should be invited to send regular statements of the persons helped by them, with the character and amount of aid given in each case. These facts, being thus collected, could be sorted, and a report returned every month to each society, showing which of its beneficiaries are assisted by others, and in what way. The plan here set forth has been carried out with complete success in Boston, Mass. The work might be limited in time to six months, from December to May in the first instance, and as an experiment for guidance in future years. We are in a position to supply the necessary details for working this plan, should it be favourably entertained.

### Alms Not the Whole of Charity.

We cannot conceal from ourselves, and would here express the conviction that alms are not the whole of charity.

Charity must do four things—

I. Relieve worthy need promptly, fittingly, and tenderly.

II. Prevent unwise alms to the unworthy.

III. Raise into independence every needy person, where this is possible.

IV. Make sure that no children grow up to be paupers.

Relief, detection, elevation, and prevention are all essential parts of a complete system.

Families or persons who have fallen into want, usually need two things—

*First.* Relief from their pressing wants—food, if hungry; fuel, if cold; or clothing, if naked. This is the work of relief.

*Secondly.* They need a long, steady, patient pull, by a wise, strong hand, up on to solid ground. This is the work which the Conference that has been assembled by Lord Randolph Churchill, and the Committee it has elected, hope to do; and a similar organisation, initiated by every Member of a metropolitan constituency, ought, in our opinion, to attempt the same, by asking the cordial co-operation of every man and woman, not only in Paddington, but every London district. It will need a combined, continuous, and prolonged effort to accomplish it, but accomplished it will be, if we determine to undertake it in the right spirit. Of course we cannot do everything at once, and for our part, we should be satisfied if our efforts for the present were confined to the collection, arrangement, and registration of the information comprised under the two heads set forth above, provided it is accompanied by the establishment of a fund raised by public subscription, for the purpose of defraying the expenses incidental to such a work as this, and the cost of providing necessary relief and assistance in such cases as may be found to require it.

### An Association of Friendly Visitors in every Constituency.

It is but fair, however, that we should state at once that we hope the present movement may ultimately result in the establishment of an Association of Friendly Workers to deal with the question of the distress in every district of London on a permanent and lasting basis. If we are to quit ourselves like men on the present occasion, success becomes a peremptory duty. All must agree that whatever methods of working amongst the poor are obstacles to complete success, should be given up in obedience to the higher law; the mere sentiment of charity is impotent, and often harmful. "Infinite talk and no work" will not suffice. We should therefore desire that an organisation, which might be called The ——— Association of Friendly Workers, should be established to bring about the following results:—

I. Thorough organisation.

II. Bringing all workers amongst the poor into co-operation.

III. Studying the whole problem and all its needs.

IV. Asking all the money needed for perfect machinery, and for sufficient relief.

V. Asking a multitude of good men and women to help in the work.

VI. Asking of a few men and women a supreme devotion.

VII. Making sure that in every varying need and phase of distress, wise, prompt, and effective measures are taken to ensure the best possible remedy.

VIII. Especially making sure that no children grow up paupers.

### The Association Constituted.

The organisation would consist of—

I. A Central Board of Directors on which representatives of all classes in each constitency should be elected.

II. A Conference of all workers and agencies amongst the poor.

III. As many "Friendly Visitors," men and women, being residents in the constituency, as may be attracted by the work, and the opportunities it affords to really help and know the poor in their immediate neighbourhood.

IV. One or more Trained and Paid Agents.

It is not necessary to go into details further now, except perhaps to explain that the Central Board of Directors might, in the first instance, be elected by the meeting of representative residents in each metropolitan constituency, assembled at the instigation of the Member representing it in Parliament. The Conference would consist of all workeıs amongst the poor of its district. It would meet every week, for at least six months in the year, and would choose an executive committee of persons interested and, as far as possible, experienced in the work. The visitors would report, usually in person, about their cases, to the Conference, which would consult and advise what action to take. The Conference should know every agency of relief, and every agent in its district. It should have in its box the card of information about every needy person in its district, which should be treated as strictly private and confidential. It should make sure that the right visitor is sent as a friend to each and every one of all these families in need. This is a vast work not yet done in any district of London, but great progress could be made in this direction during the next six months, and it ought not to be too sanguine a view to think that in five years, even this grand consummation may be attained in every metropolitan constituency.

### HOW THEY MANAGE IN PADDINGTON.

One of the first steps towards doing more for our poor fellow-citizens is to acquire a knowledge of what is already done, that our efforts may be from the first directed to the best objects, and by the speediest methods, without interference with existing organisations; and also that from these we may learn sometimes how to proceed, and sometimes what to avoid. The former is the pleasanter task, and as the metropolis possesses at least one parish in which charity is distributed according to a sound and reasonable system, it may be of value to narrate how that essential work is done in St. Paul's, Paddington. Although describing the district, however, for the sake of convenience, by the name of the parish church, we must premise that we shall speak not only of the work done by the Church of England, but of that taken up by the Westbourne Park Chapel, under the ministry of the Rev. Dr. Clifford.

In fact, the two cannot be disunited, for these two clergymen work into each other's hands, and call to their aid the Roman Catholic priest of the district, and the ministers of other denominations. They recognise as the first law of the Christian brotherhood of helpfulness, that it must be entirely unsectarian. As want and hunger know no distinction of creed, charity must take note of none. There are distinctions which it must observe, if it is to be helpful and not demoralising; but one of these is *not* whether the applicant for help belongs to one religion or another, or to none at all. Also, the clergy are in communication with the poor law guardians, that they may know when any applicant is in receipt of parochial relief, reserving to themselves the discretion of supplementing the assistance thus given if they see fit. This union in good works is a necessary precaution against charities overlapping. It is well known that the idle and unprincipled will prefer to belong to several churches, and even condescendingly give an intermittent attendance on all, if thereby they can persuade each to afford them subsistence. They profit by promiscuous, uninvestigating charity; but their profit is a loss to the community, not merely in the money they secure, to the deprivation of more deserving cases, but in the absolute demoralisation of their character, a demoralisation which is often hereditary. Paupers' children tend to grow up paupers.

The scheme now used in connexion with St. Paul's Church has been in operation since 1877, and ten years' trial has proved it to be eminently satisfactory. It is worked by the vicar, Mr. Cowell, and his assistant clergy, aided by a mission woman and an efficient staff of district visitors. It is obvious that in a parish whose population numbers 5,200, the visitors must be numerous; it is necessary also that they should be acute and discreet, knowing how to distinguish real from simulated distress. The parish clergy have, as such, the right to visit every inhabitant of the parish, and they avail themselves of it to such an extent as is necessary; that is, they become acquainted with all new-comers; and the names of these, along with the details of their position, are reported at the monthly meeting of the relief committee by the district visitors. If among the needy there are those who belong to other communions, it is sought in the first place to obtain help from those who distribute the alms of their own sect; but should this fail, the relief committee may, if on investigation the case seems to demand it, give what aid is necessary. The help granted is of various kinds—money, food, clothing, surgical appliances, recommendations for convalescent homes, school and club fees. It will therefore be seen that it covers every possible form of want, and avoids the great danger involved in giving money alone.

All applicants for relief are registered. A note is made of their names and other personal matters; the reason they require help, and the nature of the help bestowed; and whether that is likely to be temporary or permanent. Thus when a fresh application is made by or on behalf of the same person or family, a reference to the register will show what relief has been already received, and under what circumstances it was stopped. This will in many cases decide whether or not it should be renewed. It is sad, but true, that there are many cases, apparently necessitous, where it is not right that help should be given from church or private charities. It is known that the poor accept private help without that pang of wounded self-respect which they feel in applying to the poor-law authorities, and it is sometimes desirable that that pang should be felt. Take the case of drunkards and their families. The knowledge that others will provide for his wife and children encourages the drunken father to persevere in his career of ruinous self-indulgence. If he brings home but little money under ordinary circumstances, he will contribute none to family necessities if others will relieve him of them. For him there must exist the harsh knowledge that no other will bear his personal responsibilities. In those not wholly abandoned that consciousness gives the strongest reason for reformation. For the others, poor-law assistance is the only resource; it exists in order that no one, deserving or undeserving, may die of want; and if its mercies are sometimes harsh, it must be remembered that it is not expedient—not possible even if it were expedient—to deviate from the law, "As ye sow, so shall ye reap."

But leaving out of question such cases as these, the committee desire to give not merely alms, but a sufficient pension to those who, from age and infirmity, are unable to earn their living. The regularity and sufficiency of the sum given are considered essential, as chance doles, variable in amount and coming from different sources, often leave the recipients in semi-starvation, while at other times they may have more than they need, a state of affairs usually ruinous to any ideas of thrift they originally possessed. The greater efficiency of the help given under the systematised charity compared with that afforded by irresponsible giving, is proved by the fact that after the new method of careful inquiry was instituted fewer persons were helped, the undeserving being eliminated; but the sum spent was as large as ever, those retained on the register receiving larger sums at more regular intervals.

Cases of sickness make the most frequent demand for temporary relief. Before helping these a doctor's opinion as to the nature and probable duration of the illness is required, and the assistance is given mostly in kind—orders for food, and, if the doctor advises it, for medical necessaries and stimulants. Though this is done ungrudgingly, however, wherever immediate help is required, the committee always strive to impress upon the other members of the sick person's family the duty of themselves making provision for the emergency, if it be at all possible. If, for instance, the father or bread-winner of the family is ill, an effort is made to provide work for the wife and such of the children as are old enough to help. Thus an attempt is made to prevent the demoralising effect on the family of being wholly dependant on charity. The poor are also impressed with the duty of providing for the possibilities of the future by joining a sick club and provident dispensary. It has been the duty of the committee sometimes to refuse help on the ground that the applicant, having been in receipt of good wages, did not then become a member of a club. In other cases the relief asked for has been given, but on the understanding that it would not be repeated if the applicant did not, on regaining work take the opportunity of joining some provident society.

On the same principle, working men, in whose trade a slack time is yearly to be expected, are not considered eligible for relief unless exceptional circumstances have existed to prevent their making some provision for the idle time when they were in work. In some instances, where real distress can be proved in the case of a man of good character, the committee have occasionally gone so far as to pay the current subscription to his club, if it is proved that he has lost his work through no fault of his own.

Besides pensioning the aged, the committee try to find work for the able-bodied poor, not only by personal influence, but by paying the expense of advertisements for them; they also help in the expenses of emigration when that seems desirable; besides sending those unfortunate creatures who have come to London, in the vain hope of finding work, back to their own districts.

Finally, the information as to the recipients of relief is kept strictly private; none but the Relief Committee know who is obtaining help—a point which counts for much in dealing with the better class of the poor, who are the most sensitive and reserved. But while all this is done for the poor, the absolute pauperism of the district is decreasing. In 1877, the first year the system was working, the number of families assisted was 295, while in 1886 it had sunk to 152. There are two causes for this decrease. Those to whom temporary relief has been granted in sufficient amount have thereby tided over times of trial, and have again become self-supporting. On the other hand, the undeserving, dreading the strict investigation of their circumstances required by the committee, have learned to refrain from asking help from them. This is the work done by St. Paul's parish, which is, moreover, a poor one, being only a branch from another. Let us now consider very briefly the work done by the wholly unendowed churches.

A Nonconformist body has not the right, as the National Church has, to consider every person within the bounds of its district its rightful charge. But, while allowing for the comparative limit thus enforced to its work, that done by the Baptist Chapel in Westbourne Park, under the Rev. John Clifford, is very similar in nature to that of Mr. Cowell and his assistants in St. Paul's. The members of the chapel number about 1,300, and these are divided, according to their places of residence, into districts supervised by elders, who again command a corps of district visitors. The elders, who act in many respects as assistant pastors, form with the minister the Relief Committee; and it is the duty of an elder on hearing—directly, or through a visitor—of any case of need in his district, to inquire at once into the circumstances, and report them at the weekly elders' meeting. On consideration of the information thus received, suitable help is given—the aged are pensioned, the sick helped, and work is found for the unemployed. So inviolably is the information regarding recipients of relief kept, that not even the deacons—other office-bearers of the church, who have special charge of the finance department—know more than the initials of the persons receiving charity.

This help is given primarily to church members and adherents, who naturally seek it from their fellow-worshippers; but beyond this there is what is called the Domestic Mission, which seeks acquaintance with those outside the membership, and often outside any religious communion. Their needs are studied and relieved in the same manner as if they were of the church. This mission gave away last year over £130. Dr. Clifford is fortunate in having among his congregation several large employers of labour, who are ready to give work to many who might otherwise drift into the ranks of paupers. One of his elders, too, is working out a scheme for colonisation in Brazil and other parts of South and Central America, which may do something to lessen the pressure of over-population; while Dr. Clifford himself believes in the practical value of home colonisation. One of the special characteristics of the system in use at this chapel is that loans are sometimes made in cases proved suitable for such assistance after investigation by two elders, who are usually chosen as the most acute business men in the eldership. By this means large sums—as much as £20 sometimes—can be given to pay arrears of rent or otherwise help a struggling family to get into smooth water. It is noteworthy that in almost all cases these loans are repaid, usually by small instalments—a fact which amply demonstrates the value of the system.

Another agency in connexion with this church is a servants' register. A home for servants out of work has been formed; anyone may go there while seeking a situation, paying only a small board, but in deserving and destitute cases different members of the congregation pay the necessary expenses till the young woman gets another situation. It is usually essayed to make each organization of the church take up the interests of its members—as, for example, the Young Men's Bible Class, which is about to pay the expenses of one of its members who has fallen into bad health, and is to be sent to Australia; but this, of course, belongs more to special charitable impulse, and has less bearing on our present purpose.

That purpose is to show how a complete register of the poor of a district may be kept, and practical help afforded to those who require and deserve it, leaving only the worthless to be supported by parish rates. It is unnecessary here to give further details of the principle on which the work of Dr. Clifford's church is carried on, as it is in all respects similar to that of the vicar's committee, with the secretary of which (Mrs. Charles) the authorities of Westbourne Park Chapel are in frequent correspondence on the subject of helping the poor. That between them much good is done must, we think, be sufficiently obvious; and it is done at almost no cost. All the labour employed being voluntary, the yearly expense of working this important scheme is only the five to ten pounds required for stationery. The time and labour spent are gladly given by each worker, as part of the debt which each man owes to his brethren in the common kinship of humanity.

This has been done in one parish of London. Why should it not be done all over the metropolis? Objectors say that London is too vast for such an organisation. Is it so? London is indeed a great entity, an enormous city; but it is likewise an aggregation of small districts, each of which can singly do the work that is done in St. Paul's, Paddington. "If each would sweep before his own door we should have a clean street," says the homely old proverb; and it indicates the method to be employed. Let each parish form its own register; from these let there be compiled one for each Parliamentary district; then, when a register of all the London poor is desired, it can easily be formed by the combination of all the district registers. If any unemployed person wishes help he can make application to the district committee, who, by the communication that will naturally arise between all the committees, will often be able to obtain work for him in other districts. This will relieve the present condition in which employers and employed, who might be mutually helpful, stand hopelessly apart; and will, by tending to free and helpful intercourse between all classes, do much to solve the social problems that perplex us every day, and to bring about that brotherhood of all mankind which is the object of all religious, and should be—alas! if it is not so!—the result of all civilisation.

Doubtless similar work is being done elsewhere than in Paddington—it would be grievous to think that it is not; and we shall be glad to hear of the charitable machinery in use in other parishes. If in any respect it is superior to the system we have here described, it is the more incumbent on those who use it to let it be known, since it is by a comparison of existing charitable organisations that the necessary knowledge will be gained for forming a central one which shall combine the best qualities of all. Every hint as to investigation and registration is of great and immediate value, in order that those who wish to help their less fortunate brethren may know how best to do so, and that London may no longer lie under the reproach of letting the deserving poor starve in the midst of its wealth.

